# Human Epidermal Growth Factor Receptor Type 2 (HER2)-Positive Metastatic Breast Cancer With HER2-Negative Conversion: A Case Report and Treatment With Cyclin-Dependent Kinase (CDK) 4/6 Inhibitor for the Luminal Type

**DOI:** 10.7759/cureus.69771

**Published:** 2024-09-20

**Authors:** Ryo Funakushi, Sayaka Kuba, Michi Morita, Momoko Akashi, Susumu Eguchi

**Affiliations:** 1 School of Medical Sciences, Nagasaki University, Nagasaki, JPN; 2 Surgery, Nagasaki University Graduate School of Biomedical Sciences, Nagasaki, JPN; 3 Breast and Endocrine Surgery, National Hospital Organization Nagasaki Medical Center, Nagasaki, JPN

**Keywords:** breast cancer, cdk4/6 inhibitor, endocrine therapy in breast cancer, her2-negative conversion, rebiopsy

## Abstract

A 37-year-old woman was diagnosed with stage IIIA, estrogen receptor (ER)-positive/human epidermal growth factor receptor type 2 (HER2)-positive breast cancer. The patient received neoadjuvant chemotherapy with epirubicin and cyclophosphamide, followed by docetaxel, trastuzumab, and pertuzumab. The surgical specimen remained ER-positive/HER2-positive. Liver metastasis was detected after the completion of postoperative adjuvant trastuzumab and pertuzumab. A liver biopsy following treatment with trastuzumab emtansine (T-DM1) and trastuzumab deruxtecan (T-Dxd) revealed HER2-negative status. Cyclin-dependent kinase (CDK) 4/6 inhibitor combination endocrine therapy has been continued for 16 months to date while maintaining tumor shrinkage. It is essential to perform a rebiopsy during treatment to optimize therapy based on the subtype.

## Introduction

Selecting breast cancer treatment depends on the expression of the estrogen receptor (ER), progesterone receptor (PgR), and human epidermal growth factor receptor type 2 (HER2); however, there are instances of subtype discordance between primary and metastatic lesions [1]. According to the clinical practice guidelines of the American Society of Clinical Oncology and the European Society of Medical Oncology, repeat biopsy is recommended to confirm disease progression and ER, PgR, and HER2 status if accessible metastases are observed [2,3]. In cases where the results differ between primary and metastatic tissues, the expert consensus is to preferentially use the ER, PgR, and HER2 status of metastases for direct therapy, provided that it aligns with the clinical context [2]. Chemotherapy with anti-HER2 is the standard treatment for HER2-positive breast cancer; however, a treatment strategy after treatment with trastuzumab deruxtecan (T-Dxd) and trastuzumab emtansine (T-DM1) has not been established. When metastases become HER2-negative, it is difficult for clinicians to decide whether to change the treatment strategy to endocrine therapy with cyclin-dependent kinase (CDK) 4/6 inhibitors for the luminal type.

Palbociclib, a CDK4/6 inhibitor, has been reported to significantly prolong progression-free survival (PFS) in hormone receptor (HR)-positive/HER2-negative advanced breast cancer (ABC) when combined with aromatase inhibitors or fulvestrant, compared with aromatase inhibitors or fulvestrant alone [4-6].

Here, we report the case of a 37-year-old woman diagnosed with HER2-positive metastatic breast cancer with short disease-free survival (DFI), liver metastasis, and HER2-negative conversion in the liver metastatic tissue. The patient was diagnosed with HER2-negative metastases by rebiopsy after T-DM1 and T-Dxd and responded to endocrine therapy with a CDK4/6 inhibitor for the luminal type.

## Case presentation

**Figure 1:**
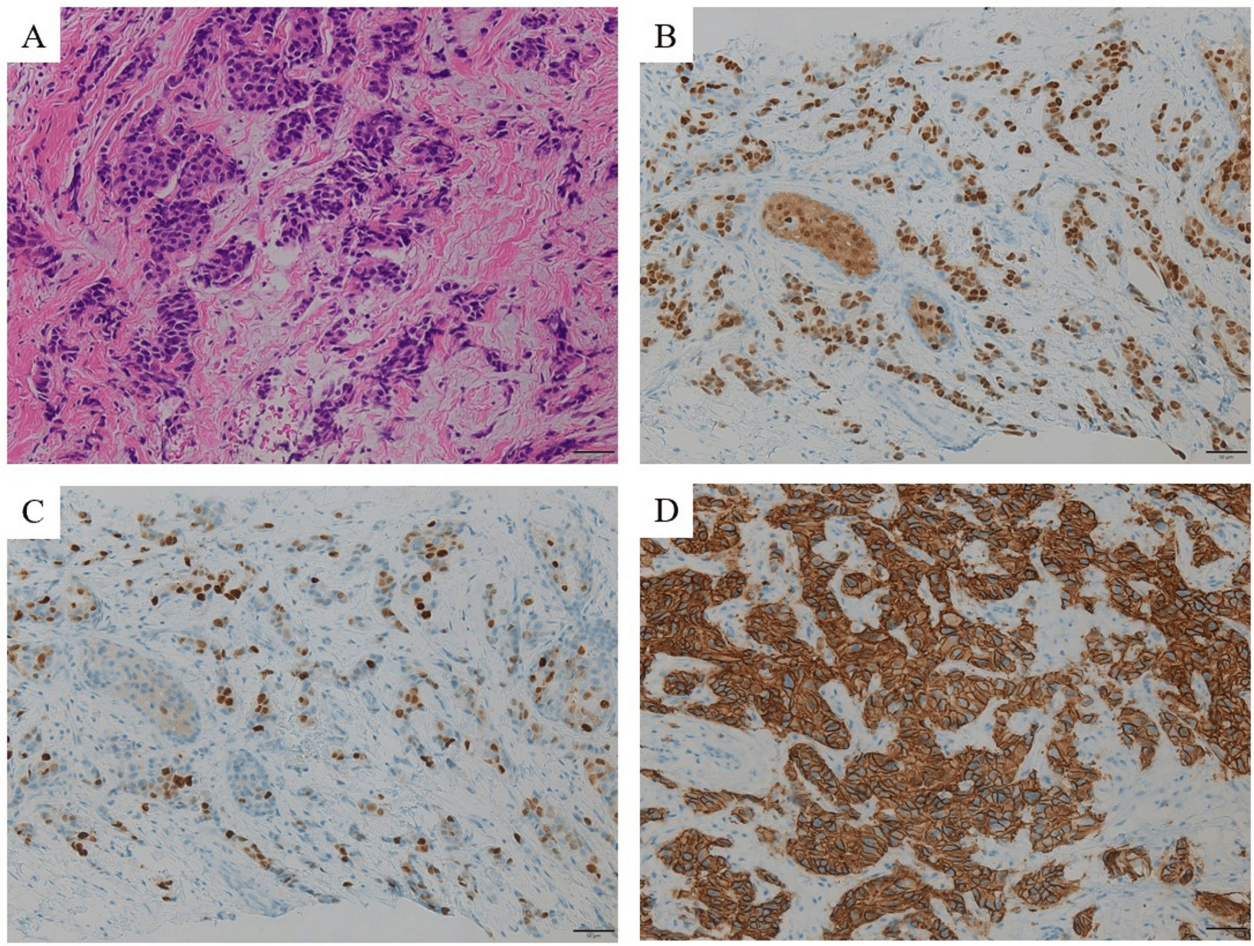
Breast core needle biopsy specimen Along with extensive fibrosis, the cells show nuclear enlargement and variability in size. There is also infiltration and proliferation of atypical cells, forming cord-like structures (H&E, A). Immunohistochemical staining of (B) ER is positive (approx. 70%), (C) PgR is positive (approx. 50%), and (D) HER2 shows strong membrane staining. H&E: hematoxylin and eosin; ER: estrogen receptor; PgR: progesterone receptor; HER2: human epidermal growth factor receptor type 2

A 37-year-old lactating woman with no family history of breast or ovarian cancer visited our clinic with a primary complaint of a right breast mass. The patient was diagnosed with breast cancer ER-positive, PgR-positive, and HER2-positive (Figures 1A-1D).

**Figure 2:**
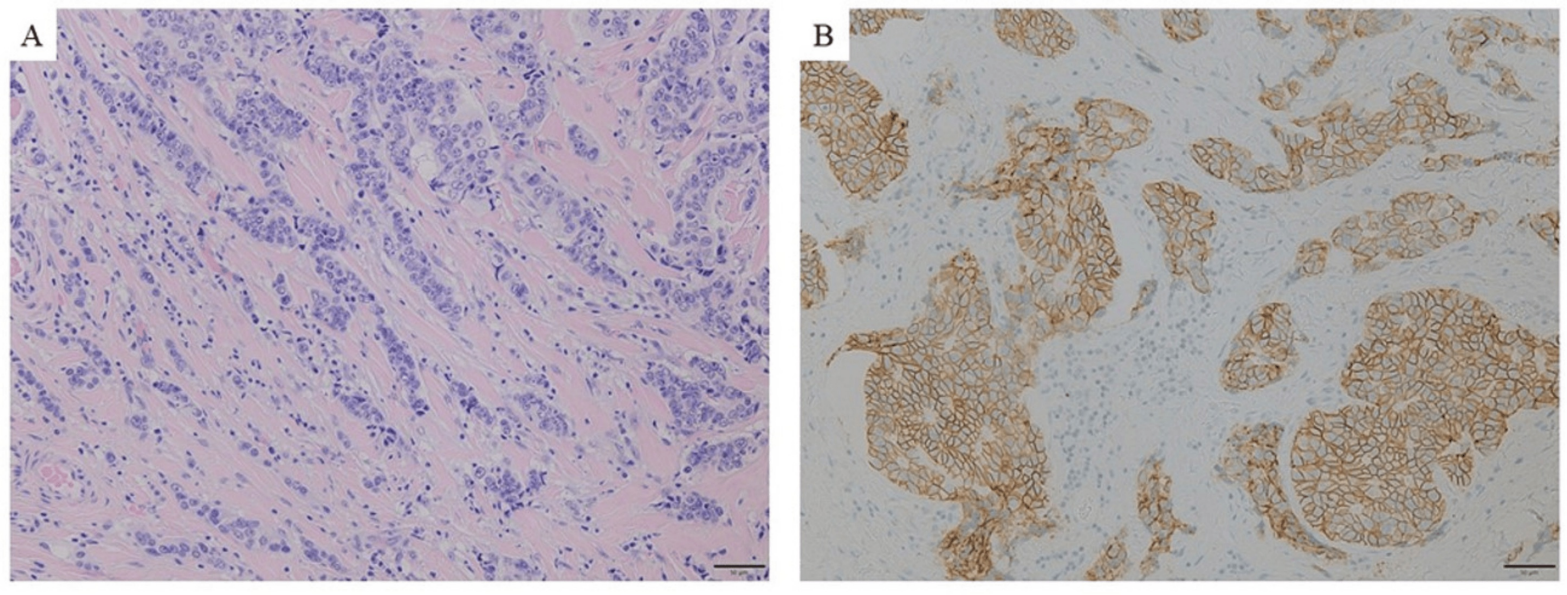
Breast surgery specimen (A) H&E staining. While fibrosis likely due to chemotherapy is observed, there is also an extensive presence of viable cancer cells. (B) HER2 staining shows strong circumferential membrane staining in >30%. H&E: hematoxylin and eosin; HER2: human epidermal growth factor receptor type 2

The patient underwent a plain computed tomography (CT) scan due to asthma, which revealed no distant metastasis. The staging was cT3N1M0, indicating cStage IIIA. The patient was treated with four cycles of epirubicin and cyclophosphamide (EC), followed by four cycles of docetaxel, trastuzumab, and pertuzumab as preoperative chemotherapy. After neoadjuvant chemotherapy, the patient underwent total mastectomy and axillary lymph node dissection. Pathological analysis revealed a tumor size of 30 × 15 × 100 mm with positive lymphatic and vascular invasion and lymph node metastasis. The histopathological response to chemotherapy was grade 0. The surgical specimen was positive for ER, PgR, and HER2, consistent with core needle biopsy performed before neoadjuvant chemotherapy (Figures 2A, 2B).

**Figure 3:**
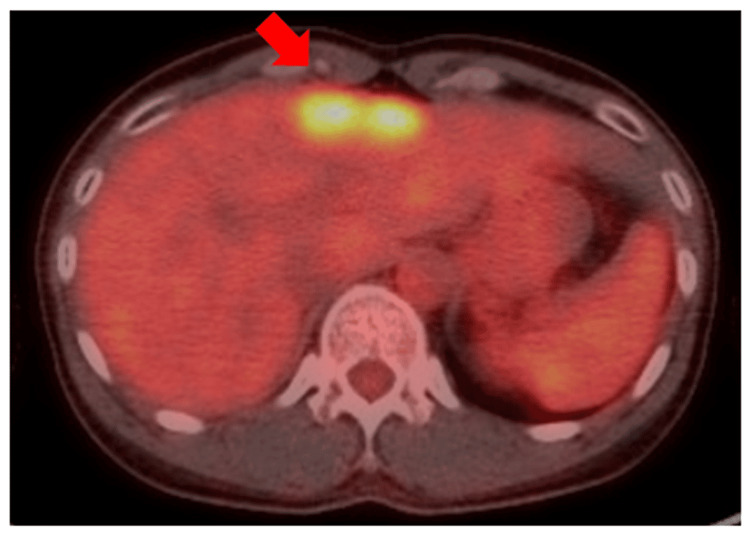
PET/CT image at the time of diagnosis of breast cancer metastases Two adjacent hotspots in the liver were diagnosed as liver metastases. PET/CT: positron emission tomography/computed tomography

As the efficacy of T-DM1 in the adjuvant setting had not been reported at the time, adjuvant therapy included post-mastectomy radiation therapy, trastuzumab, pertuzumab, luteinizing hormone-releasing hormone (LHRH) agonists, and tamoxifen. After completing trastuzumab and pertuzumab treatment and continuing endocrine therapy in the clinic, the patient returned to our hospital because of an elevation in the tumor marker carbohydrate antigen (CA) 15-3. Positron emission tomography/computed tomography (PET/CT) revealed high fluorodeoxyglucose uptake at two locations in the liver, leading to the diagnosis of a metastatic liver tumor (Figure 3).

**Figure 4:**
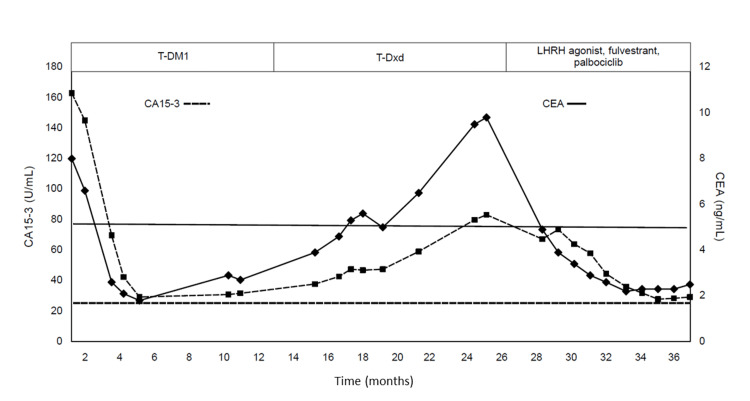
Transition of tumor markers (CEA and CA15-3) after starting T-DM1 therapy Tumor markers were elevated at the time of liver metastasis. They decreased with T-DM1 treatment but progressively increased during T-Dxd treatment. In contrast, they decreased with CDK4/6 inhibitor treatment. CA15-3: carbohydrate antigen 15-3; CEA: carcinoembryonic antigen; LHRH: luteinizing hormone-releasing hormone; T-Dxd: trastuzumab deruxtecan; T-DM1: trastuzumab emtansine; CDK4/6: cyclin-dependent kinase 4/6

She subsequently underwent T-DM1 therapy; her tumor marker levels decreased and the tumor shrank. However, 10 months later, the cancer grew (Figure 4).

Subsequently, we changed the antitumor agent from T-DM1 to T-Dxd. The tumor marker levels gradually increased, and the tumor continued to increase progressively.

**Figure 5:**
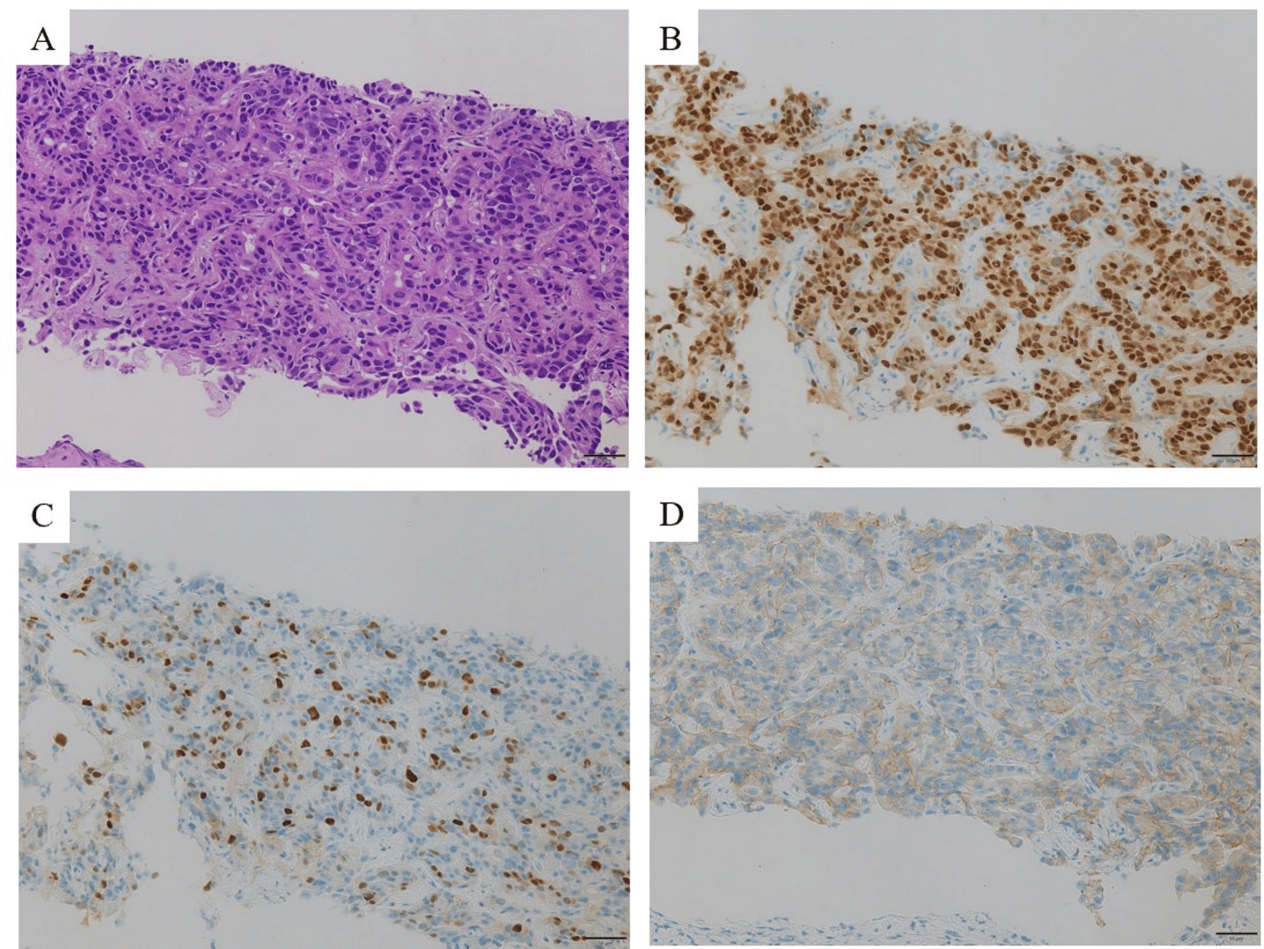
Liver biopsy specimen (A) H&E staining. Tumor cells with a high nucleo-cytoplasmic ratio, similar to those in primary breast cancer, are proliferating in a cord-like fashion with associated fibrous stroma. Immunohistochemical staining of (B) ER, (C) PgR, and (D) HER2. ER and PgR are positive, but HER2 shows weak circumferential membrane staining in >10%, and therefore assessed as 2+. H&E: hematoxylin and eosin; ER: estrogen receptor; PgR: progesterone receptor; HER2: human epidermal growth factor receptor type 2

The best overall outcome with T-Dxd was only in stable disease with gradual tumor growth, but treatment was continued for 13 months. A liver biopsy was performed to confirm the subtype and for gene panel testing. The tumor was positive for ER and PgR; however, it was HER2 2+ and judged HER2-negative as the luminal type without amplifying the dual-color in situ hybridization (Figure 5).

Gene panel testing revealed PIK3CA mutations, and alpelisib, CDK4/6, or mammalian target of rapamycin (mTOR) inhibitor were recommended. In Japan, alpelisib is not available through public insurance. Treatment with the LHRH agonists fulvestrant and palbociclib gradually decreased the levels of carcinoembryonic antigen (CEA) and CA15-3 tumor markers and magnetic resonance imaging (MRI) showed shrinkage of the tumor (Figure 4). The patient has been continuing treatment with LHRH agonist, fulvestrant, and palbociclib for 16 months to date.

## Discussion

CDK4/6 inhibitors have improved progression-free and overall survival compared to aromatase inhibitors alone in patients with HR-positive/HER2-negative ABC. Here, we report the successful treatment of HR-negative/HER2-positive primary tissue and HR-positive/HER2-negative metastatic disease after T-DM1 and T-Dxd treatment using a CDK4/6 inhibitor. This is the first report on the efficacy of endocrine therapy with CDK4/6 inhibitors in patients with ABC who exhibited HER2-negative conversion upon rebiopsy.

New HER2-targeted drugs, including antibody-drug conjugates (ADCs), have been developed and approved for HER2-positive breast cancer; continuing anti-HER2 therapy after disease progression has become the standard treatment [7]. However, loss of HER2 expression in metastatic tumors can occur in patients with primary HER2-positive breast cancer [1,8-10]. In a previous study, patients whose metastatic HER2 status changed to negative had shorter overall survival than those whose metastatic HER2 status remained positive [8]. One reason this subtype changes during treatment is the heterogeneity of breast cancer cells [11,12]. Dual HER2 blockade with lapatinib and trastuzumab in HER2-positive disease induces a low proliferative luminal A phenotype in tumors from neoadjuvant-treated patients and in vitro models [13]. It is unclear whether treatment should be changed according to metastatic subtype [9,13].

Another resistance mechanism to anti-HER2 therapy includes the abnormal activation of the phosphoinositide 3-kinase (PI3K)/protein kinase B (AKT) pathway induced by PTEN loss, PIK3CA mutations, and the retention of cyclin D1 expression after anti-HER2 therapy [14,15]. Palbociclib selectively inhibits CDK4 and CDK6 activity by inhibiting cyclin D and CDK4/6 complex activity, preventing retinoblastoma (Rb) phosphorylation, and arresting the transition from the gap 1 (G1) to synthesis (S) phase in the cell cycle. CDK4/6 inhibitors have been reported to effectively increase the luminal phenotype following anti-HER2 therapy in vitro effectively [13]. The Palbociclib Ongoing Trials in the Management of Breast Cancer (PALOMA)-2 and PALOMA-3 phase III trials showed that palbociclib combined with endocrine therapy resulted in almost twice the PFS compared with endocrine monotherapy in patients with HR-positive/HER2-negative ABC [4,5]. In contrast, in patients with HER2-positive tumors, CDK4/6 inhibitors and anti-HER2 therapy have been reported to directly suppress tuberous sclerosis complex (TSC) 2 phosphorylation, mTOR and S6 ribosomal protein (S6RP) functions, and Rb phosphorylation, thus demonstrating antitumor effects [16]. MonarcHER, a phase II trial in women with HR-positive/HER2-positive ABC who had previously received at least two HER2-targeted therapies for ABC, reported that a three-drug combination of abemaciclib, trastuzumab, and fulvestrant significantly prolonged PFS compared to standard-of-care chemotherapy plus trastuzumab [17]. CDK4/6 inhibitors for HER2-positive ABC require further investigation; however, CDK4/6 inhibitors should be considered if metastases are rebiopsied and HER2-negative conversion occurs during treatment.

## Conclusions

In conclusion, various anti-HER2 therapies are available that are effective against HER2-positive breast cancer. However, if tumors develop resistance to anti-HER2 therapy, conducting a rebiopsy and considering a treatment strategy switch from anti-HER2 therapy based on the subtype of the metastatic lesions should be contemplated.
